# Magnetic Nitrogen-Doped Porous Carbon Nanocomposite for Pb(II) Adsorption from Aqueous Solution

**DOI:** 10.3390/molecules26164809

**Published:** 2021-08-09

**Authors:** Fatimah Mohammed Alzahrani, Norah Salem Alsaiari, Khadijah Mohammedsaleh Katubi, Abdelfattah Amari, Abubakr M. Elkhaleefa, Faouzi Ben Rebah, Mohamed A. Tahoon

**Affiliations:** 1 Chemistry Department, College of Science, Princess Nourah Bint Abdulrahman University, Riyadh 11671, Saudi Arabia; fmalzahrani@pnu.edu.sa; 2 Department of Chemical Engineering, College of Engineering, King Khalid University, Abha 61411, Saudi Arabia; amelkhalee@kku.edu.sa; 3 Research Laboratory of Energy and Environment, Department of Chemical Engineering, National School of Engineers, Gabes University, Gabes 6072, Tunisia; 4 Higher Institute of Biotechnology of Sfax (ISBS), Sfax University, P.O. Box 263, Sfax 3000, Tunisia; benrebahf@yahoo.fr; 5 Department of Chemistry, College of Science, King Khalid University, P.O. Box 9004, Abha 61413, Saudi Arabia; tahooon_87@yahoo.com; 6 Chemistry Department, Faculty of Science, Mansoura University, Mansoura 35516, Egypt

**Keywords:** magnetic nanocomposites, N-doped porous carbon, water treatment, adsorption, Pb^2+^ removal

## Abstract

We report in the present study the in situ formation of magnetic nanoparticles (Fe_3_O_4_ or Fe) within porous N-doped carbon (Fe_3_O_4_/N@C) via simple impregnation, polymerization, and calcination sequentially. The synthesized nanocomposite structural properties were investigated using different techniques showing its good construction. The formed nanocomposite showed a saturation magnetization (M_s_) of 23.0 emu g^−1^ due to the implanted magnetic nanoparticles and high surface area from the porous N-doped carbon. The nanocomposite was formed as graphite-type layers. The well-synthesized nanocomposite showed a high adsorption affinity toward Pb^2+^ toxic ions. The nanosorbent showed a maximum adsorption capacity of 250.0 mg/g toward the Pb^2+^ metallic ions at pH of 5.5, initial Pb^2+^ concentration of 180.0 mg/L, and room temperature. Due to its superparamagnetic characteristics, an external magnet was used for the fast separation of the nanocomposite. This enabled the study of the nanocomposite reusability toward Pb^2+^ ions, showing good chemical stability even after six cycles. Subsequently, Fe_3_O_4_/N@C nanocomposite was shown to have excellent efficiency for the removal of toxic Pb^2+^ ions from water.

## 1. Introduction

Human activities and development depend essentially on water [1,2]. Water pollution and associated problems are increased with the rapid progress of urbanization, industrialization, and the direct release of various contaminants such as heavy metals into clean water sources [3,4]. The hard degradation of heavy metals, their persistence, toxicity, and mobility in water sources have made these ions the most dangerous of all contaminants. Heavy metal ions have an acute effect on the health of human and marine organisms even at very low concentrations due to their cumulative effect, making their existence in water very hazardous [5]. Lead (Pb^2+^) ions have been deemed typical and characteristic inorganic contaminants among all heavy metal ions [6,7]. Refuse incineration, metallurgy, mineral exploration, lead-acid batteries, and the manufacturing industry are the main sources of Pb^2+^ pollutants [8]. The permitted limit of Pb^2+^ ions in drinking water is <50 μg/L [9]. The safety of biosystems and the public health are in real danger when the Pb^2+^ concentrations in water sources are above the limit of 5.0 μg/L fixed by the E.U. for human water consumption [10]. Several methods have been developed for the removal of heavy metal from water, including adsorption [11,12,13], lime softening [14], ion exchange [15], membrane separation [16], coagulation [17], and precipitation [18]. Among all these methods, the adsorption process is considered the most applicable method for heavy metal removal due to low processing cost, high efficiency, and safety [19,20,21]. However, the application of adsorption in water treatment faces a major problem related to the difficulty in separating the adsorbent from the aqueous medium after treatment. Thus, the improvement of an easily separated, reusable, and efficient adsorbent for the capture of heavy metals from water is a great challenge. In recent years, nanomaterials (NMs) [22,23,24], as a unique class of materials, are widely used as adsorbents for the removal of heavy metals and other pollutants. Interestingly, among all NMs, carbon NMs are applied for the adsorption of various pollutants due to their low cost, ease of synthesis, and high surface area [25]. As mentioned above, the problem of the adsorbent separation appeared especially for carbon NMs due to their hydrophilicity and adaptability [26]. Herein, magnetic separation offers fast and effective separation of the adsorbent from the treatment environment when compared to centrifugation and filtration. Thus, the magnetic nanoparticles (such as iron oxide nanoparticles) must be impeded with the carbon NMs for separation purposes. Besides the separation purpose, iron oxide nanoparticles (Fe_3_O_4_ NPs) have good adsorption properties, are ecofriendly with naturally abundant properties, and are low cost. The synergetic effect of Fe_3_O_4_ NPs and carbon NMs could improve their adsorption properties toward several pollutants as reported recently. Moreover, the coating of Fe_3_O_4_ NPs with carbon NMs increases their stability. This combination between magnetic NPs and carbon NMs was previously reported for the removal of different pollutants from water [27,28,29]. Moreover, the interaction and the adsorption capacity of carbon NMs can be improved by adding heteroatom (such as nitrogen, sulfur, and oxygen) to their structure. Heteroatom doping is associated with the development of new technologies such as electrocatalysts and supercapacitors [30,31] due to the doping effect on the optical, electronic, and structural properties of carbon NMs. The use of doped carbon NMs for adsorption of pollutants from water was reported by several studies [32,33,34]. The adsorption affinity and selectivity of carbon NMs toward different pollutants (organic or inorganic) can be modified by doping heteroatoms into the carbon lattice. It is reasonable to think that the adsorption properties of doped carbon NMs are different from those of non-doped materials. Motivated by all of the above, magnetic Fe_3_O_4_/nitrogen-doped porous carbon nanocomposite (Fe_3_O_4_/N@C) using rice husk as carbon precursor was synthesized using simple impregnation, polymerization, and calcination. The synthesized nanocomposite was characterized using different techniques and examined for the removal of Pb^2+^ ions from water. Additionally, Fe_3_O_4_/N@C nanocomposite showed excellent adsorption behavior toward Pb^2+^ ions. Finally, the adsorption mechanism between Pb^2+^ ions and Fe_3_O_4_/N@C nanocomposite was determined via the study of adsorption kinetics and isotherms.

## 2. Results and Discussions

### 2.1. The Characterization of Fe_3_O_4_/N@C Nanocomposite

**Figure 1:**
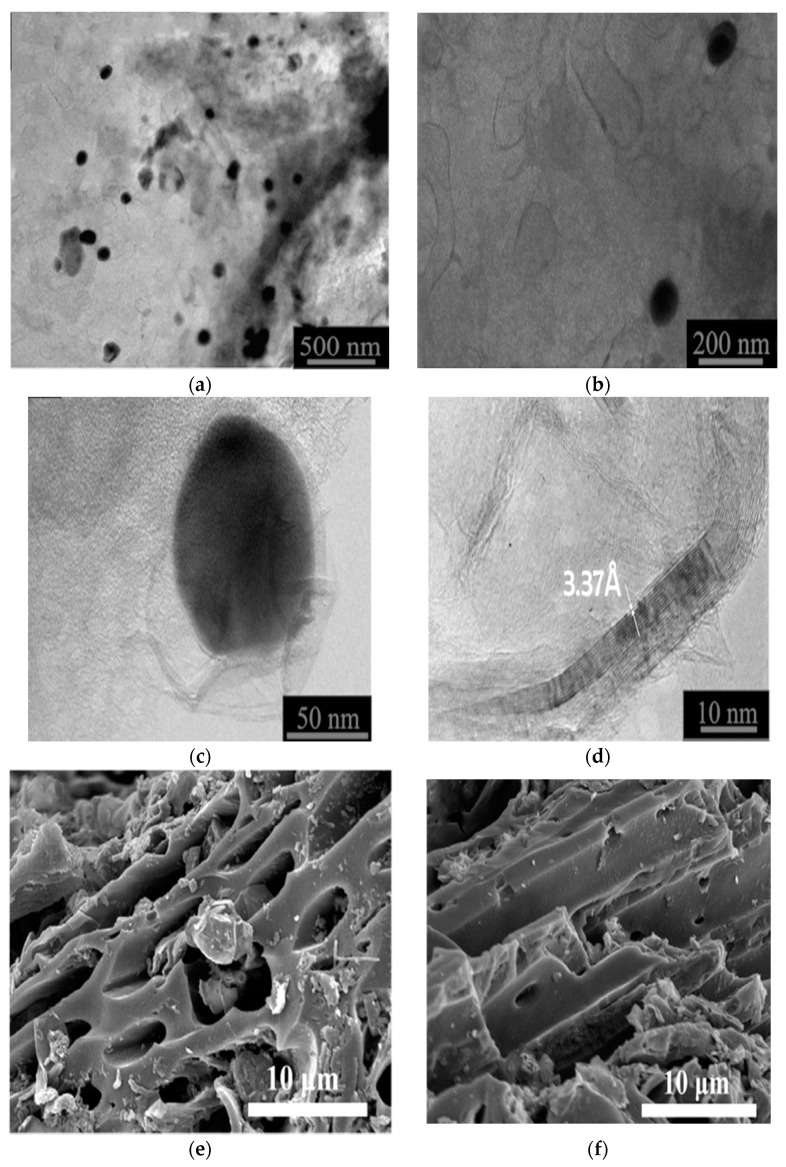
TEM images (**a**–**d**) and SEM images (**e**,**f**) of Fe_3_O_4_/N@C nanocomposite.

For the evaluation of the surface morphology of Fe_3_O_4_/N@C nanocomposite, TEM images were provided as shown in Figure 1a–d. According to Figure 1a, the magnetic nanoparticles (Fe_3_O_4_) are well distributed over the surface of the framework (N@C). The average particle size of the nanoparticles over the matrix was equal to 47.5 nm as revealed from Figure 1a,b. Some capsules were detected at higher magnification (Figure 1b). Additionally, Figure 1c shows the magnification image of a randomly selected nanoparticle. Figure 1c indicates the presence of thin layers that wrap the nanoparticles. This layer thickness is about 5.0 to 10.0 nm. Moreover, according to Figure 1d, the graphite-like structure has a thickness of <10.0 nm, an interplanar distance of 3.37 Å, and involves 5.0 to 20.0 graphene layers. Since such thin layers are formed in amorphous carbon when calcined with iron oxide, the observed layers are most likely the result of the calcination of polymerized pyrrole.

**Figure 2:**
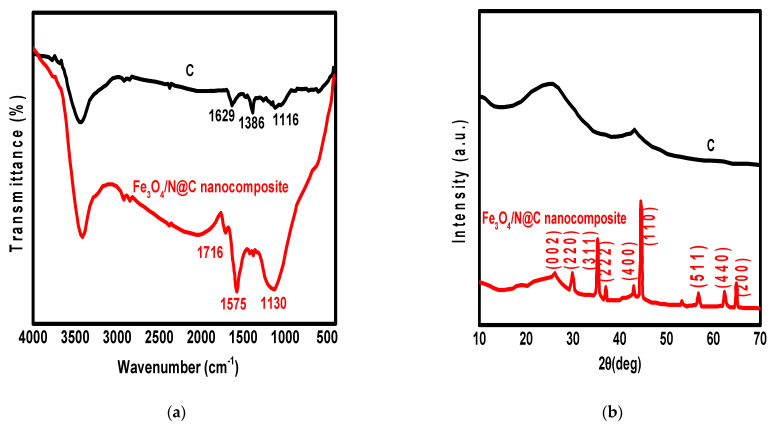

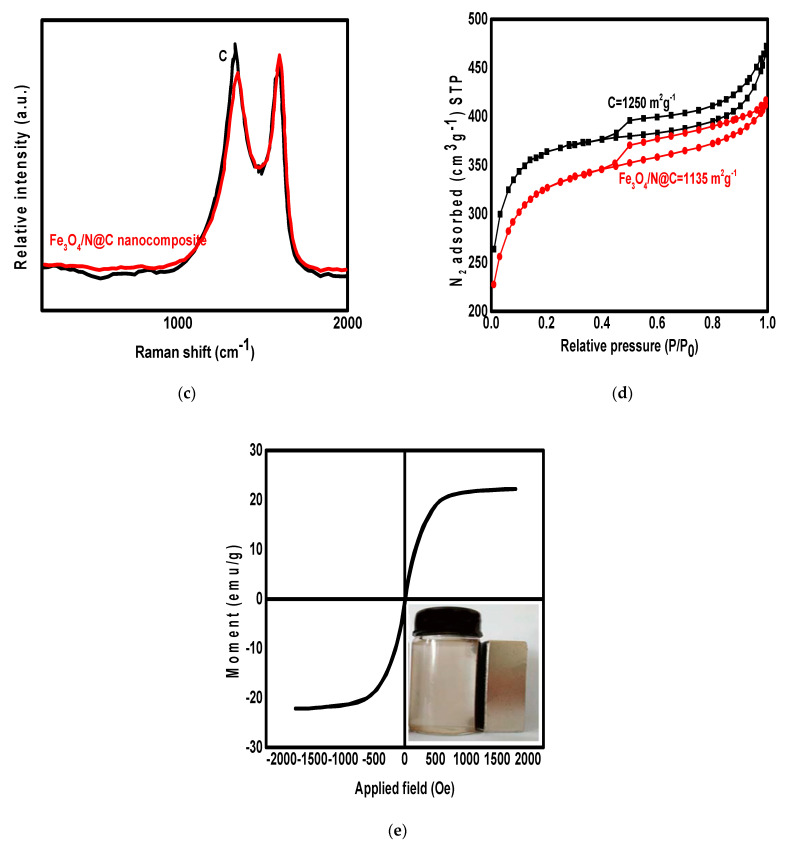
FT-IR (**a**), XRD (**b**), Raman shift (**c**), N_2_ adsorption isotherms (**d**) of rice husk carbon and Fe_3_O_4_/N@C nanocomposite, and magnetization curve of Fe_3_O_4_/N@C nanocomposite (**e**) (inset: the magnetic separation of the nanocomposite using an external magnet after Pb^2+^ adsorption).

For further morphology investigation of granulated Fe_3_O_4_/N@C nanocomposite, SEM images were provided as shown in Figure 1e,f. Figure 1e shows the cross-view while Figure 1f shows the side view of the tunnel-like structures. The matrix clearly contains mesopores and macropores as shown in Figure 1e,f. The matrix clearly has a large surface area for the adsorption of Pb^2+^ ions resulting from the hierarchical 3-D and homogeneous parts as shown in SEM images. For the determination of the functional groups present in N@C and its magnetic nanocomposite (Fe_3_O_4_/N@C), FT-IR spectra were provided as shown in Figure 2a. According to Figure 2a, the FT-IR bands of N@C at 1575 cm^−1^ and 1716 cm^−1^ represent asymmetric stretching vibrations of -COO- and C=O, respectively. There are differences between the two spectra of N@C and Fe_3_O_4_/N@C, which are represented by: (i) the new band at 1386 cm^−1^ that is attributed to the stretching vibration of C-N bond; (ii) the band at 1629 cm^−1^ that is attributed to stretching vibrations of C=C or in-plane deformation vibrations of N-H bond; and (iii) the calcination which caused the disappearance of the stretching band of C-H at 2922 cm^−1^. Additionally, the XRD pattern of N@C and Fe_3_O_4_/N@C nanocomposite was taken to investigate their structural properties (Figure 2b). According to Figure 2b, there are differences between the XRD of N@C and Fe_3_O_4_/N@C represented by the appearance of the Fe_3_O_4_ new diffractions at 2θ = 62.6, 57.0, 43.2, 35.5, and 30.2 corresponding to (440), (511), (400), (311), and (220) planes, respectively [35]. The existence of patterns at 2θ = 65.0 and 44.8 are attributed to the elemental Fe planes (200) and (110), respectively. This elemental Fe may be produced in the matrix during the calcination process. Furthermore, the XRD of N@C showed a broad peak (2θ = 26.5), characteristic of its amorphous structure; this broad peak became obvious in the XRD of the nanocomposite Fe_3_O_4_/N@C and matched with 0.35 nm of an interlayer d spacing, showing the similarity of reported bulk CN materials [36] with the present graphite-like materials in the graphitic ordering. The average particle size of Fe_3_O_4_ is 45.0 nm, calculated using the Scherrer equation, depending on the pattern at 35.5. There is matching between the particle size measured through the TEM image (47.5 nm) and the value calculated from XRD. The graphite-like structures were formed and this can additionally be proven by using Raman spectra as shown in Figure 2c. The degree of material ordering can be interpreted using the ratio between two Raman bands (I_D_/I_G_) [37]. The creation of graphite-like structures with a well-defined sp^2^ hybridized carbon was approved since the I_D_/I_G_ ratio was 1.088 for N@C, higher than that of Fe_3_O_4_/N@C nanocomposite, which is equal to 0.913.

**Figure 3:**
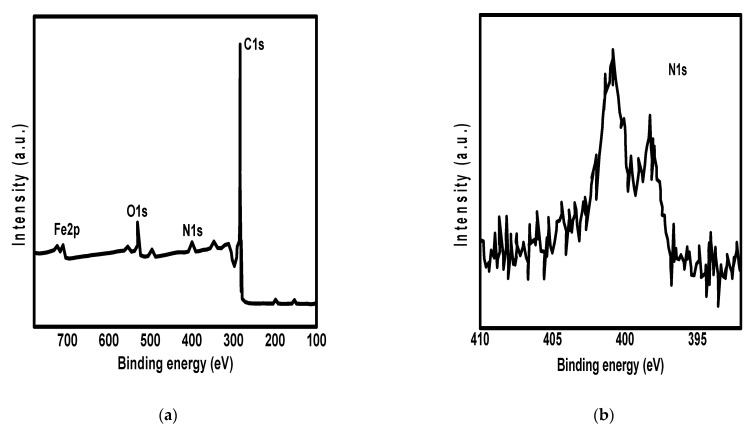
XPS full survey spectra (**a**) and survey for nitrogen (**b**) of the synthesized Fe_3_O_4_/N@C nanocomposite.

For the investigation of pore size distribution and specific surface area of N@C and Fe_3_O_4_/N@C nanocomposite, Brunauer–Emmett–Teller (BET) method was used through nitrogen adsorption-desorption measurements as shown in Figure 2d. According to Figure 2d, N@C and Fe_3_O_4_/N@C nanocomposite show hysteresis loops in the range of 0.4 to 1.0 (P/P_O_) with 3.9 nm of a pore size distribution, indicating that the mesopores are connected inside the nanocomposites. Moreover, the specific surface area was high for Fe_3_O_4_/N@C nanocomposite (1135 m^2^·g^−1^), which is attributed to their porous nature. The specific surface area for N@C (1250 m^2^·g^−1^) was higher than that of Fe_3_O_4_/N@C nanocomposite, indicating the decrease of the surface after the modification with the Fe_3_O_4_. This reduction of the surface area is familiar as the materials’ pores are blocked with the introduction of functional groups. However, the specific surface area is not responsible for the adsorption capacity of the nanosorbent. For example, specific surface areas of MM, SM, and CM adsorbents prepared by Tuutijarvi et al. [38] were 203.2, 90.4, and 51.0 m^2^/g, respectively, while their adsorption capacities toward arsenic ions were in the order: CM > SM > MM. This indicates that the functional groups increase the chelation capacity of the adsorbent toward different pollutants. So, the high specific surface area and the mesopores of the synthesized Fe_3_O_4_/N@C nanocomposite benefit the chelation of Pb^2+^ ions from an aqueous solution. Moreover, a vibrating sample magnetometer (VSM) was used for the determination of magnetic properties of Fe_3_O_4_/N@C nanocomposite and the magnetization curve was observed in Figure 2e. According to Figure 2e, the Fe_3_O_4_/N@C nanocomposite showed a saturation magnetization (M_s_) equal to 23 emu·g^−1^ at room temperature with a weak hysteresis indicating that the magnetic nanoparticles were near to the superparamagnetic. After the adsorption of Pb^2+^ ions on the surface of Fe_3_O_4_/N@C nanocomposite, an external magnet was used to collect the nanocomposite in a few seconds as shown in Figure 2e inset. This rapid response of the magnetic adsorbent to the external magnetic field facilitates its separation from aqueous solution after Pb^2+^ ions removal as well as the easy reuse of the nanocomposite for water treatment several times. The Fe_3_O_4_/N@C nanocomposite’s elemental map was recognized via using the XPS (Figure 3). Figure 3a shows the XPS full survey for the nanocomposite that fulfilled the expectations that it would contain the peaks of oxygen (O1s), iron (Fe 2p^3^), nitrogen (N1s), and carbon (C1s).

Figure 3b shows the XPS survey for nitrogen that displays two binding energy peaks for N1s. The first peak that represents sp^2^ N atoms attached to carbon atoms appears at the lower binding energy (398.0 eV). The second one representing N atoms triagonally attached with carbon atoms (sp^2^ or sp^3^) appears at the higher binding energy (400.5 eV).

### 2.2. The Adsorption Properties

#### 2.2.1. The Optimization of Adsorption Conditions

**Figure 4:**
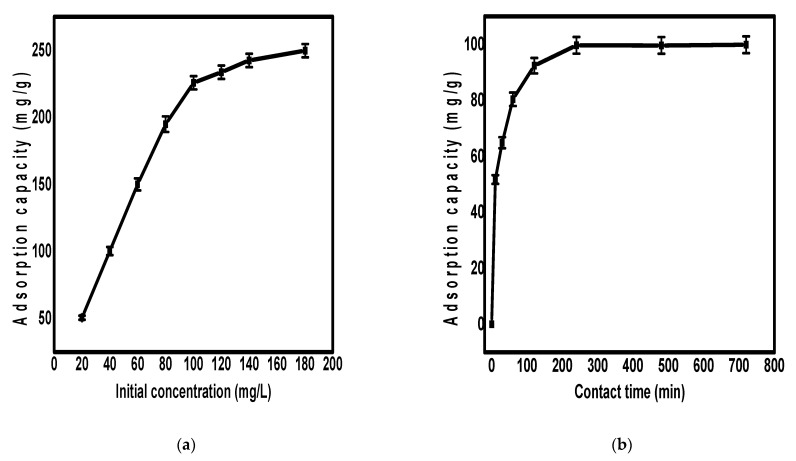

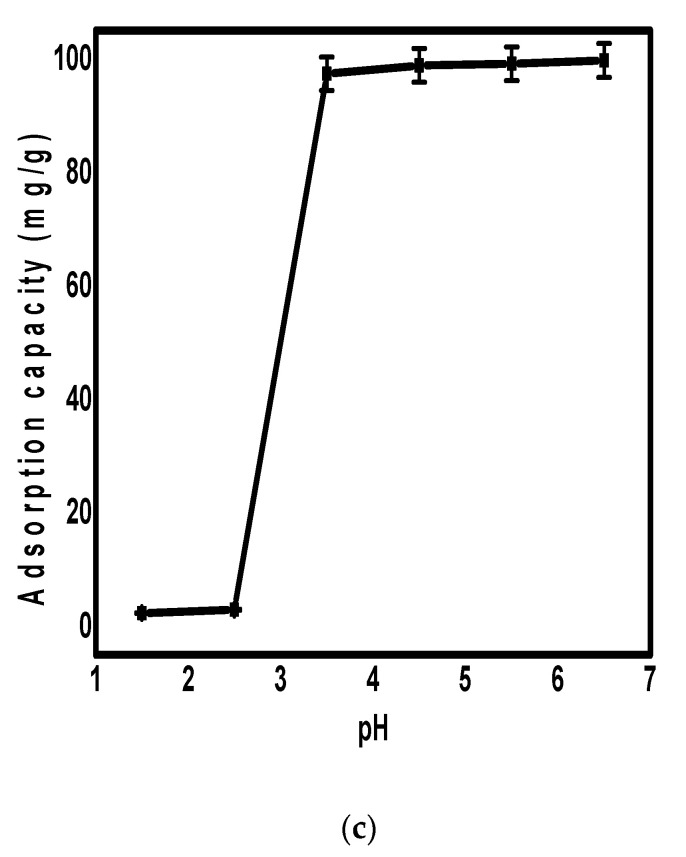
The effect of pH value (**a**), contact time (**b**), and initial concentration (**c**) on the adsorption of Pb^2+^ ions on the surface of Fe_3_O_4_/N@C nanocomposite.

The adsorption efficiency is determined through the study of the effect of different operating conditions, including initial concentration effect, contact time, and pH. The optimization of such parameters helps to achieve the best adsorption results for the studied adsorbent. Accordingly, the effect of these conditions on the uptake of Pb^2+^ ions using Fe_3_O_4_/N@C nanocomposite was studied as shown in Figure 4. The effect of the initial concentration of Pb^2+^ ions was studied in the range of 20.0 mg/L to 180.0 mg/L while maintaining the other parameters as constant (Figure 4a). According to Figure 4a, the adsorbent high surface area saved the vacant active adsorption sites to chelate the Pb^2+^ ions for concentration up to 100 mg/L, and this behavior is very clear from the linear increase of the adsorption capacity in the range of 20.0 mg/L to 100.0 mg/L. The adsorption capacity increase showed a slower increasing rate above the concentration of 100.0 mg/L that could be attributed to the decrease in the number of vacant adsorption sites by the gradual increase in the number of Pb^2+^ ions. When the nanocomposite was examined for the adsorption of Pb^2+^ ions at a concentration of <20.0 mg/L, a removal efficiency more than 91% was observed, indicating its potential use for water treatment even at low concentrations. The effect of contact time on the adsorption of Pb^2+^ ions on the surface of Fe_3_O_4_/N@C nanocomposite was studied and the results are shown in Figure 4b. According to Figure 4b, the first hour showed a rapid increase in the adsorption capacity. After 5.0 h, the Pb^2+^ adsorption rate became slower until saturation was reached.

The most important factor affecting the adsorption process is known to be the pH solution. Therefore, the effect of the pH value on the adsorption of Pb^2+^ ions on the surface of Fe_3_O_4_/N@C nanocomposite was studied in the pH range of 1.0 to 6.5 as shown in Figure 4c. According to Figure 4c, the maximum adsorption capacity was reached at pH 6.5. The adsorption capacity showed a small increase when the pH increased from 3.5 to 6.5 at which the maximal value was obtained. At pH value < 3.5, the adsorption capacity showed a sharp drop to 3.0 mg/g which is attributed to the competition between H^+^ ions and Pb^2+^ for the adsorption sites on the surface of the nanocomposite. Of course, this competition will be decided for H^+^ ions due to their smaller weight. Moreover, the adsorbed H^+^ ions on the surface of the adsorbent can cause repulsion with the Pb^2+^ ions and therefore, a drop in their adsorption on the materials. The pH effect results indicated the ability to reuse the nanocomposite for the removal of Pb^2+^ ions several times by the substitution of the adsorbed Pb^2+^ ions by H^+^ ions.

#### 2.2.2. Adsorption Kinetics

**Figure 5:**
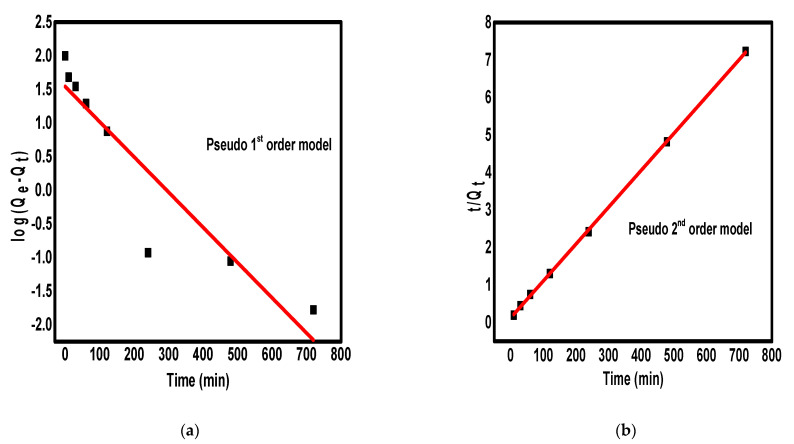
The pseudo-first-order kinetic model (**a**) and pseudo-second-order kinetic model (**b**) for the adsorption of Pb^2+^ ions on the surface of Fe_3_O_4_/N@C nanocomposite.

The two familiar kinetics models pseudo-first-order and pseudo-second-order were used to fit the adsorption experimental results for more understanding of the mechanism of adsorption. The pseudo-first-order and pseudo-second-order models are given according to Equations (1) and (2), respectively.

log(Q_e_ − Q_t_) = log(Q_e_ − (k_1_/2.303)t) (1)

t/Q_t_ = (1/k_2_Q_e_^2^) + (t/Q_e_) (2)

where the symbols k_1_, k_2_, Q_e_, and Q_t_ denote the pseudo-first-order rate constant (min^−1^), the pseudo-second-order rate constant (g/mg/min), the amount of metal ions adsorbed at equilibrium, and the amount of metal ions adsorbed at a time (t, min), respectively. The linearized plots of pseudo-first-order and pseudo-second-order are shown in Figure 5a,b respectively, and the kinetic parameters are given in Table 1.

According to the correlation coefficients (R^2^) in Table 1, the experimental results fit more with the pseudo-second-order model (R^2^ = 0.9998) than the pseudo-first-order model (R^2^ = 0.8490), indicating that the removal of Pb^2+^ ions on the surface of Fe_3_O_4_/N@C nanocomposite occurred via a chemisorption mechanism in which the nanocomposite and Pb^2+^ ions shared the electrons [39,40,41]. Additionally, the chemisorption mechanism of Pb^2+^ ions uptake by Fe_3_O_4_/N@C nanocomposite indicated that the adsorption rate is controlled by the number of vacant sites unoccupied by metal ions.

Subsequently, we can interpret the adsorption of Pb^2+^ ions over Fe_3_O_4_/N@C nanocomposite surface as follows: the metal ions surrounded the outer surface of the nanocomposite by diffusion followed by the diffusion of Pb^2+^ ions into the inner surface of the nanocomposite and the adsorbent inner surface finally attached the metallic ions. The adsorption rate could be affected by any step of the metal ions uptake into the adsorbent inner surface.

#### 2.2.3. Adsorption Isotherm

**Figure 6:**
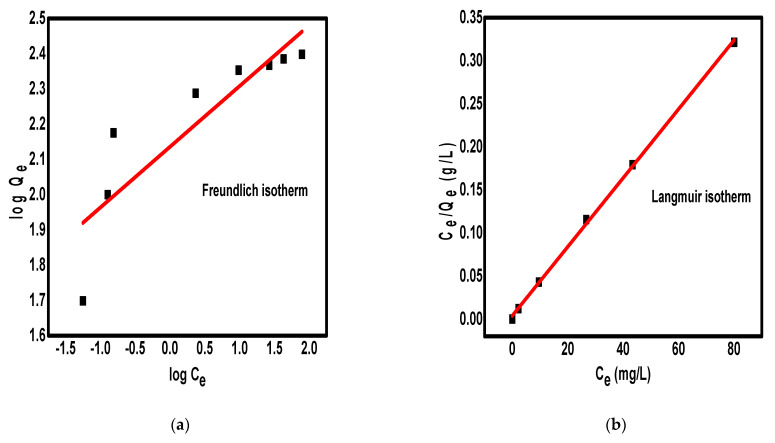
The Freundlich isotherm model (**a**) and Langmuir isotherm model (**b**) for the adsorption of Pb^2+^ ions on the surface of Fe_3_O_4_/N@C nanocomposite.

To analyze the adsorption of Pb^2+^ ions on the surface of Fe_3_O_4_/N@C nanocomposite correctly, Freundlich and Langmuir isotherm models were used as shown in Figure 6a,b, respectively. The Freundlich and Langmuir models can be represented as Equations (3) and (4), respectively [42,43].

Q_e_ = K_f_C_e_^(1/n)^ (3)

Q_e_ = (K_L_Q_m_C_e_)/(1+K_L_C_e_) (4)

The symbols Q_e_, C_e_, n, and K_f_ denote the adsorption capacity at equilibrium (mg/g) and the equilibrium concentration of metal ions (mg/L), adsorption intensity, and adsorption capacity constants, respectively. Q_m_ is the maximum adsorption capacity (mg/g) while K_L_ is the Langmuir equilibrium constant. Equations (3) and (4) can be represented by their linearized form according to Equations (5) and (6), respectively.

logQ_e_ = logK_f_ + (1/n) logC_e_ (5)

1/Q_e_ = (1/Q_m_) + (1/K_L_Q_m_) · (1/C_e_) (6)

The Freundlich isotherm model is well known, assuming the heterogeneous adsorption of adsorbate ions on the surface of the adsorbent through energetically asymmetrical adsorption sites [44,45]. In contrast, the Langmuir isotherm model assumes the monolayer and homogeneous adsorption of ions through energetically identical sites [46,47,48]. The Freundlich and Langmuir parameters are calculated and shown in Table 1. The applicability of the isotherm model was compared by judging correlation coefficients (R^2^) values. The value of R^2^ was 0.7331 for the Freundlich isotherm and 0.9991 for the Langmuir isotherm. This suggested that the adsorption data fit better with the Langmuir model than the Freundlich model. This means that the adsorption of Pb^2+^ ions on the surface of Fe_3_O_4_/N@C nanocomposite occurred through monolayer adsorption [49]. Moreover, this indicates that all sites over the surface of the synthesized nanosorbent are energetically identical and the uptake of Pb^2+^ ions from aqueous solution is homogeneous [50]. According to the Langmuir model, the maximum adsorption capacity of Fe_3_O_4_/N@C nanocomposite for Pb^2+^ ions was found to equal 250.0 mg/g. This indicates that the adsorption of Pb^2+^ ions occurs at specific adsorption sites on the adsorbent until the adsorption capacity reaches a saturation state [51]. Thus, the Fe_3_O_4_/N@C nanocomposite showed a very high adsorption capacity toward the studied metal ions. This high adsorption capacity could be attributed to many reasons, such as the presence of magnetic nanoparticles (Fe_3_O_4_) that may improve the adsorption capacity of Pb^2+^ ions via iso-electronic substitution [52]; the abundant porous structure of Fe_3_O_4_/N@C nanocomposite and its high surface area; and the presence of nitrogen (sp^2^-hybridized) lone pair electron that is also available to donate the vacant orbitals of metallic ions. Moreover, the iron crystal lattice contains hydroxyl ions that can be replaced by the Pb^2+^ ions with the avoidance of crystal structure disturbance [53]. It is clear that the structural properties of the synthesized porous Fe_3_O_4_/N@C nanocomposite could enhance its ability for removal of positively charged cations from water and must be examined soon for the removal of additional cations and cationic dyes.

### 2.3. Comparison of Fe_3_O_4_/N@C Nanocomposite with other Adsorbents

The adsorption performance of Pb^2+^ ions onto Fe_3_O_4_/N@C nanocomposite was compared with other adsorbents as listed in Table 2. According to Table 2, the Q_m_ of Fe_3_O_4_/N@C nanocomposite for Pb^2+^ adsorption is 250.0 mg/g, which is significantly higher than other adsorbents. As listed in Table 1, most of the described adsorbents display a limitation in adsorption capacity for Pb(II) removal which ranges from 30.0 to 233 mg/g.

We deduce from this comparison that the present porous Fe_3_O_4_/N@C nanocomposite is a promising nanosorbent for the removal of Pb^2+^ ions from water and must be investigated soon for the removal of additional pollutants.

### 2.4. Reusability Study

**Figure 7:**
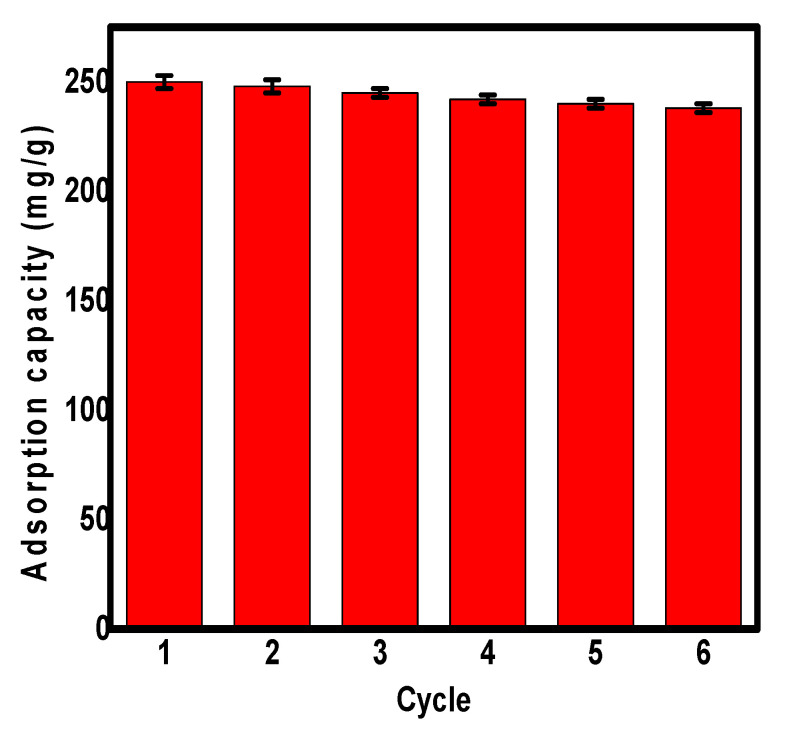
Pb^2+^ ions removal on the surface of recycled Fe_3_O_4_/N@C nanocomposite up to six successive cycles.

Regeneration and recycling are of important significance for the application of any sorbent in the treatment of real water samples [64,65]. After each adsorption cycle of Pb^2+^ ions over the Fe_3_O_4_/N@C nanocomposite, 1.0 M HCl was used as an eluent for the effective desorption of metallic ions. After each adsorption cycle, the nanosorbent was collected from the aqueous solution using an external magnetic field, without the need to apply the filtration method, due to the magnetic properties of the synthesized adsorbent. The Fe_3_O_4_/N@C nanocomposite can be regenerated and reused as investigated up to six successive cycles with a minor decrease of the initial adsorption capacity as shown in Figure 7. According to Figure 7, the adsorption capacity for the removal of Pb^2+^ ions decreased after six cycles only by 5.0% of the initial adsorption capacity. This showed the chemical stability of the synthesized nanocomposite after six cycles. Moreover the excellent reusability results indicated the ability to reuse Fe_3_O_4_/N@C nanocomposite as adsorbent for water treatment several times, which is important from an economic point of view due to the decrease of the treatment cost.

## 3. Materials and Methods

### 3.1. Chemicals

Rice husk collected from the countryside of Mansoura City, Dakhlia Province, Egypt was chosen as a precursor for the preparation of porous carbon. Inorganic impurities and adhering dust were removed from the rice husk by washing several times with tap water then by deionized water. Potassium hydroxide (KOH, 99%), ferric chloride (FeCl_3_·6H_2_O), and lead nitrate (Pb(NO_3_)_2_) were purchased from Sigma-Aldrich. Hydrochloric acid (HCl) and sodium hydroxide (NaOH) were purchased from Al-Nasr Co., Egypt. All chemicals were analytical grade and were used as received without any modification.

### 3.2. Synthesis of Fe_3_O_4_/N@C Magnetic Nanocomposite

The Fe_3_O_4_/N@C magnetic nanocomposite was synthesized typically according to the next brief steps which are optimized in a preliminary study to determine the best conditions for the nanocomposite synthesis. Firstly, hierarchical structures of porous carbon were synthesized using rice husk as a precursor through carbonization at 650 °C. A tubular furnace (KOYO, Tokyo, Japan) was used for the carbonization under the continuous flow of N_2_ (120 mL/min) for 4.0 h. Then, the activation process was achieved using an alkaline solution (KOH) as an activator at 700 °C. KOH and as-prepared rice husk carbon were mixed and placed in the tubular furnace at 700 °C with the continuous flow of nitrogen (150 mL/min) for 2.0 h. After that, distilled H_2_O was used to wash the carbon material until stable pH was achieved, followed by the drying process for 4.0 h in the hot air oven (80.0°C) to obtain the activated carbon. Then, the activated carbon was ground into fine granules followed by mixing granulated carbon (0.50 g) with ferric chloride solution (0.417 g, 0.9M) for 2.0 h. This mixture was milled and ultrasonicated until it reached a dense paste. After that, pyrrolization of the paste was performed by exposure to pyrrole vapor for 1.0 h at 50.0 °C in a closed vessel that allows the migration of pyrrole to the pores of carbon, and the achievement of polymerization of pyrrole resulted from Fe^3+^ catalytic effect. This pyrrolization method is simple, cheap, and provides high nitrogen content. Finally, the calcination of the paste was performed under N_2_ atmosphere for 2.0 h at 850.0 °C. After that, the synthesized Fe_3_O_4_/N@C nanocomposite became ready for characterization and application.

### 3.3. Characterization

The synthesized Fe_3_O_4_/N@C nanocomposite gained via using N-porous carbon derived from rice husk (N@C) as a matrix (framework) for the magnetic nanoparticles was characterized using different familiar characterization techniques such as transmission electron microscope (TEM), scanning electron microscope (SEM), Fourier-transform infrared spectroscopy (FT-IR), X-ray diffraction (XRD), Raman spectroscopy, X-ray photoelectron spectra (XPS), and vibrating sample magnetometer (VSM). TEM analysis was carried out at an accelerating voltage of 200.0 kV using FEI Tecnai F20 transmission electron microscope while SEM analysis was carried out at 15.0 kV using a JEOL JSM-6360LV field emission microscope. FT-IR spectra were obtained via KBr pellet technology using a Vector 22 FTIR spectrometer. XRD analysis was achieved using Cu K_α_ radiation (λ = 0.15406 nm) by Bruker D8 Focus diffractometer at a scanning rate of 5.0° min^−1^. The Raman microscope (Renishaw inVia Qontor) was used to measure Raman spectra. XPS spectra were performed using an X-ray source of Mg K radiation on a spectrometer, Physical Electronics PHI 5400. Lakeshore 7407 vibrating sample magnetometer (VSM) was used to measure the magnetic properties. ASAP2020 volumetric adsorption analyzer was used to perform nitrogen adsorption studies at 77.0 K.

### 3.4. Batch Adsorption Experiment

Batch experiments were used to study pH effect, adsorption kinetics, and adsorption isotherms for the removal of Pb^2+^ ions over the synthesized Fe_3_O_4_/N@C nanocomposite. A stock solution of Pb^2+^ ions (200.0 mg/L) was prepared and then diluted to get any required concentration for the experimental study. The effect of pH was studied in the range of 1.5 to 6.5 using 0.10 M of NaOH and HCl to adjust the pH value. During the pH effect study, the temperature used was 25.0 °C, Pb^2+^ initial concentration was 40.0 mg/L, the solution volume was 50.0 mL, and the adsorbent dosage was 20.0 mg. The same conditions were used to study the adsorption kinetics and isotherms, except the initial Pb^2+^ concentration during the isotherm study ranged from 20.0 mg/L to 200.0 mg/L. During the kinetics and isotherms study, the pH was adjusted at 3.5, corresponding to the optimum pH value. In all cases, the mixture was shaken for 12.0 h at 120.0 rpm until it reached equilibrium. After each study, the magnetic nanocomposite was separated using an external magnet and the remaining solution was examined for the presence of Pb^2+^ ions using ICP.

The adsorption capacity at equilibrium (Q_e_) can be calculated using the following equation:

Q_e_ = (C_o_ − C_e_)V/m (7)

The symbols m, V, C_e_, and C_o_ denote the mass of adsorbent (g), the volume of the solution (L), equilibrium concentration (mg/L), and initial concentration (mg/L), respectively.

## 4. Conclusions

Herein, a simple impregnation followed by polymerization and calcinations succeeded in the synthesis of a magnetic nanocomposite (Fe_3_O_4_/N@C) designed from Fe_3_O_4_ nanoparticles and porous N-doped carbon derived from rice husk as a raw material. The nanocomposite was structurally characterized using different techniques, including TEM, SEM, FT-IR, XRD, Raman spectroscopy, VSM, and XPS. The results indicated the good construction of the nanocomposite. The capturing of Pb^2+^ ions was enhanced due to the increased negative charge density that resulted from the presence of doped N atom in the porous carbon as confirmed by XPS results. Due to the complexation reaction between Fe_3_O_4_ or N lone pair electrons with the Pb^2+^ ions as well as the high surface area of activated carbon, the synthesized nanocomposite showed fast kinetics and high adsorption capacity toward the toxic Pb^2+^ ions. The adsorption of Pb^2+^ ions on the surface of Fe_3_O_4_/N@C followed a pseudo-second-order kinetic model and Langmuir isotherm. According to Langmuir isotherm, the nanocomposite showed a maximum adsorption capacity equal to 250.0 mg/g. The superparamagnetic properties of the synthesized nanocomposite enabled the fast collection of the adsorbent using an external magnet instead of classical filtration, which encouraged the investigation of adsorbent reusability. The reusability study indicated the chemical stability of the nanocomposite up to six cycles, with a minor loss in the adsorption capacity. Thus, the Fe_3_O_4_/N@C nanocomposite is a promising nanosorbent for the removal of Pb^2+^ ions from water.

## Figures and Tables

**Table 1:** The adsorption isotherm and kinetic parameters for the adsorption of Pb^2+^ ions on the surface of Fe_3_O_4_/N@C nanocomposite.

Item	Coefficient
Pseudo 1st order	R^2^ = 0.8470
	k_1_ (min^−1^) = 0.14
	Q_e_ (mg/g) = 34.70
Pseudo 2nd order	R^2^ = 0.9998
	k_2_ (mg/g/min) = 7.885 × 10^−3^
	Q_e_ (mg/g) = 101.88
Freundlich isotherm	R^2^ = 0.7331
	n = 5.90
	K_f_ (mg/g)(L/mg)^(1/n)^ = 135.0
Langmuir isotherm	R^2^ = 0.9991
	K_L_ (L/mg) = 1.540
	Q_max_ (mg/g) = 250.0

**Table 2:** Comparison of Fe_3_O_4_/N@C nanocomposite with other adsorbents for the adsorption of Pb^2+^ ions.

Adsorbent	pH Value	Removal Capacity (mg/g)	Ref.
Fe_3_O_4_/N@C	5.5	250.0	This study
Highly pure biosilica	5.0	120.5	[54]
HCl-treated Egyptian kaolin	5.5	34.5	[55]
Modified beer lees	4.0	29.6	[56]
CCN	5.0	232.5	[57]
Geopolymer-alginate-chitosan	5.0	142.67	[58]
ZnO nanoparticles	6.0	114.9	[59]
Carbon nanotubes/Fe_3_O_4_–NH_2_	5.30	75	[60]
Fe_3_O_4_@silica–xanthan	5.0	24.3	[61]
Carbon nitride	4.0	65.6	[62]
Carbon nanotubes	5.0	37.4	[63]

## Data Availability

Not applicable.

## Sample Availability

Not available.
